# Development of Perovskite (MACl)_0.33_FA_0.99_MA_0.01_Pb(I_0.99_Br_0.01_)_3_ Solar Cells via n-Octylammonium Iodide Surface Passivation

**DOI:** 10.3390/nano13091492

**Published:** 2023-04-27

**Authors:** M. M. Osman, A. M. El-naggar, A. Q. Alanazi, A. M. Aldhafiri, A. A. Albassam

**Affiliations:** 1 Research Chair of Exploitation of Renewable Energy Applications in Saudi Arabia, Physics & Astronomy Department, College of Science, King Saud University, P.O. Box 2455, Riyadh 11451, Saudi Arabia; 2 Physics Department, Faculty of Science (Boys Branch), Al-Azhar University, Cairo 11884, Egypt; 3 National Center for Renewable Energy Technology, KACST, P.O. Box 2455, Riyadh 11442, Saudi Arabia; aqalanazi@kacst.edu.sa; 4 Physics & Astronomy Department, College of Science, King Saud University, P.O. Box 2455, Riyadh 11451, Saudi Arabia

**Keywords:** photovoltaic, perovskite, solar cells, surface passivation, optical, n-octylammonium, structure, AFM, FTIR, PL

## Abstract

The influence of n-octylammonium iodide (OAI, passive layer) on the types of phases formed in a (MACl)_0.33_FA_0.99_MA_0.01_Pb(I_0.99_Br_0.01_)_3_ perovskite film was studied using X-ray diffraction. Using UV spectrophotometric techniques, it was determined how varied OAI additive layer ratios affected the linear and nonlinear optical characteristics of glass substrates/FTO/compact TiO_2_/mesoporous TiO_2_/(MACl)_0.33_FA_0.99_MA_0.01_Pb(I_0.99_Br_0.01_)_3_ films. All films’ direct optical bandgap energies were determined to be 1.54 eV. The effects of OAI addition on the films’ photoluminescence intensity and emitted colors were also investigated. For the fabricated perovskite solar cells (PSCs) without an OAI passivation layer, the corresponding power conversion efficiency (PCE), open-circuit voltage (V_OC_), short-circuit current density (J_SC_), and fill factor (FF) values were 18.8%, 1.02 V, 24.6 mAcm^−2^, and 75%, respectively. When the concentration of OAI reached 2 mg, the maximum obtained values of PCE, V_OC_, J_SC_, and FF were 20.2%, 1.06 V, 24.2 mAcm^−2^, and 79%, respectively. The decreased trap density and increased recombination resistance were responsible for the improvement in solar cell performance.

## 1. Introduction

Due to their ease of fabrication, organic–inorganic hybrid halide perovskites materials have emerged as effective low-cost materials for high-power-conversion-efficiency solar cells [1,2]. Hybrid halide perovskite materials exhibited improved optoelectronic properties [3], a tunable bandgap [4], a high visible light absorption coefficient [5], and a long diffusion length of photogenerated charge carriers [6]. Perovskite solar cells (PSCs) have improved their power conversion efficiency (PCE) from 3.8% [7] to 25.7% [8], but their PCE, open-circuit voltage (Voc), and fill factor (FF) are still below the theoretical values [9,10]. The performance of the devices was enhanced by improving the bulk and interface quality of the perovskite.

The nonradiative recombination rate of the charge carriers is more important than a radiative recombination mechanism in PSCs [11,12]. The nonradiative recombination occurs via shunt pathways between ETL and HTL lyres, the hole transport layer (HTL) interface, the electron transport layer (ETL) interface, and the bulk of the perovskite. In addition, the quick growth of the perovskite crystal causes defects within the structure of the perovskite materials. These defects help to create a nonradiative carrier recombination site [13]. Furthermore, there is recombination within the lattice defects created within the perovskite materials because they have energy levels inside the band gap (near the conduction or valence bands). The major carrier loss mechanism was found to occur at the interfaces between the perovskite and transport layers. As a result, the interfaces between the layers of charge transport and perovskite are passivated [14].

Chemical passivation decreases the energy states of defects, and the process that separates defects spatially from minority charge carriers in the device is called physical passivation [15]. The processes used in the chemical passivation methods include additive incorporation, dimensional engineering, fabrication engineering, composition engineering, and interface adaptation [15]. In the field of photovoltaics, the “dimensional engineering method” is efficient and attracting much interest. According to dimensional engineering, ABX_3_ perovskite can be made into one-dimensional (1D) or two-dimensional (2D) crystal structures by incorporating large organic cations into its conventional 3D structure. As a result, a mixed 1D–3D or 2D–3D perovskite structure can be produced, increasing the stability and efficiency of the device’s light-conversion process [15].

High efficiency and long-term stability must be guaranteed for solar cells to be commercially successful. On the other hand, PSCs made of 3D perovskites have a low stability despite having a high efficiency [6,16]. Many studies have found that, compared to 3D PSCs, 2D PSCs exhibit substantially greater environmental sustainability [17,18,19,20]. As a result of the limited efficiency of PSCs based entirely on a 2D structure, mixed perovskite containing both 3D and 2D PSCs have been the subject of intense research [21,22,23,24,25,26,27,28,29,30,31,32,33,34,35]. A 3D/2D graded structure has been used to increase the stability and efficiency of PSCs [21,26,28,29,30,31,32,33,35]. The primary benefits of 2D on 3D include defect passivation on the perovskite’s surface and an increase in hydrophobicity due to the alkylammonium cations that form the 2D layer. A chloroform or isopropanol (IPA) solution of 1D and 2D cations can be deposited after a 3D perovskite photoactive layer to create a layer covered in 1D/2D perovskite. A 1D/2D perovskite film is produced on top of the 3D perovskite by the mixed-cation passivation layer [28,36]. A long-chained phenylethylammonium (PEA^+^), octylammonium (OA+), methylammonium (MA^+^), butylammonium (BA^+^), and cyclopropylammonium (CA^+^) are typically used as spacer cations [15]. Due to the hydrophobic properties of alkylammonium cations, researchers have incorporated them to passivate defects and increase ambient stability [37,38].

The sandwiched structure of PSCs includes an n-type electron transport layer (ETL) and a hole transport layer (HTL) [39,40]. Although perovskite employed as an absorber is almost neutral, it is typically thought of as slightly p-type [41,42,43]. A p–n junction structure is seen in silicon solar cells. The p–n junction’s charge depletion separates the electron from the hole. Drift-moving carriers have greater mobility and lifetimes than diffusion-moving carriers. Because perovskite exhibits an insulator-like behavior and has a low intrinsic carrier concentration, the ETL/perovskite/HTL junction in PSCs is an n–i–p junction. The ETL/perovskite junction has a strong electric field bias, whereas the HTL/perovskite junction has a low electric field bias. In this regard, the existence of a highly dielectric 2D layer between HTL and perovskite will impact the bilateral electric field, which can enhance charge transfer and reduce carrier recombination. Consequently, even though it is predicted that defect passivation of the 3D surface depending on the type of alkyl that the alkylammonium cation forming the 2D layer will be similar, it is thought that there will be a change in the electric field due to the change in electrical properties caused by the difference in the dipole moment. Finally, in addition to the defect passivation, changes in humidity stability and charge separation and transport may result from the type of alkylammonium cation that forms the 2D layer on the 3D surface.

In this work, we used a large cation of OAI to demonstrate successful interface modification between the perovskite layer and HTL in PSCs. OAI has been proven to enhance the efficiency of PSCs by chemical passivation of the perovskite layer and producing an internal barrier that prevents charge collection from the perovskite layer. Furthermore, their hydrophobic properties resulted in an increase in the device’s stability.

## 2. Methods and Materials

**Figure 1:**
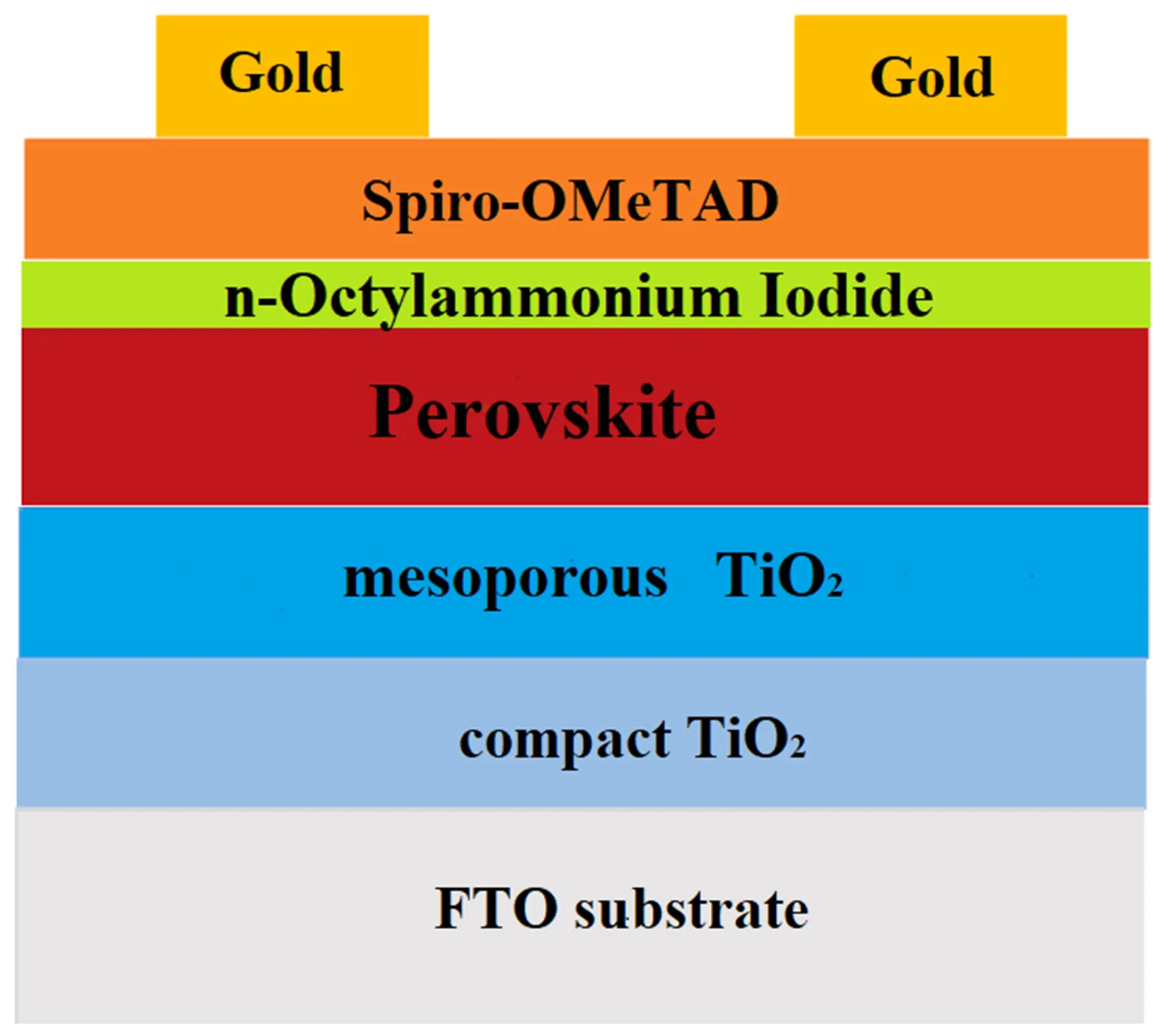
Device structure of the OAI-passivated PSC.

The devices were fabricated by fluorine-doped tin-oxide (FTO)-coated glass substrates etched using the laser etching method. FTO glass substrates were cut into 2.5 cm * 1.7 cm. After that, the substrates were cleaned for 30 min by soaking and sonicating in 2% Hellmanex III in deionized water, deionized water, acetone, and ethanol, respectively. The cleaned substrates were treated with UV ozone for 30 min prior to processing. A compact TiO_2_ (c-TiO_2_) layer was deposited as the ETL layer by the spray pyrolysis method at 450 °C. More information can be obtained in [44]. A mesoporous TiO_2_ (m-TiO_2_) layer was deposited by the spin coating method as shown in [44]. The precursor solution of perovskite (MACl)_0.33_FA_0.99_MA_0.01_Pb(I_0.99_Br_0.01_)_3_ was prepared as shown in [45]. Different concentrations of n-octylammonium iodide (2, 4, and 6 mg) were dissolved in 1 mL of isopropyl alcohol to be used as a passivation layer. The passivation layer was deposited on the perovskite layer (after cooling down) by spinning the solution (70 μL) at 6000 rpm for 30 s (acceleration rate: 2000 rpm/s^2^). The passivated perovskite films were coated with a Spiro-MeOTAD and gold layers as shown in [45]. Figure 1 shows the device structure of the OAI-passivated PSC.

The film thickness was measured using the DEKTAK profilometer. The thickness of all films was approximately the same at 500 ± 10 nm. Using X’Pert Pro (MRD) from Panalytical (the goniometer has a minimum 2-step size of 0.0001°), the structure of the prepared films was determined. The surface morphology and topography of the passivated perovskite layer were investigated using an atomic force microscope (AFM) (NanoScope V from Bruker SPMs). Fourier transform infrared (FTIR) spectra were recorded using an infrared spectrometer (Spectrum Two FTIR, PerkinElmer). Time-resolved photoluminescence (TRPL) was measured via time-correlated single-photon counting (TCSPC) using a LifeSpec II (Edinburgh Instruments) fluorescence spectrometer with a picosecond pulsed diode laser (EPL-510, Edinburgh Instruments) at a 510 nm wavelength and an 85 ps pulse width. A photoluminescence (PL) system with a 532 nm excitation laser source was used to obtain the PL spectra (described in [44]). The transmittance (T) and reflectance (R) spectra of the prepared films were investigated using a double-beam spectrophotometer (Cary5000 from Varian, 200–3200 nm). Using a Cell Tester system with a Model No. CT50AAA from Photo Emission Tech, the I–V characteristics were measured with a device area of 0.158 cm^2^ and an irradiation of 1000 W/m^2^.

## 3. Results and Discussion

**Figure 2:**
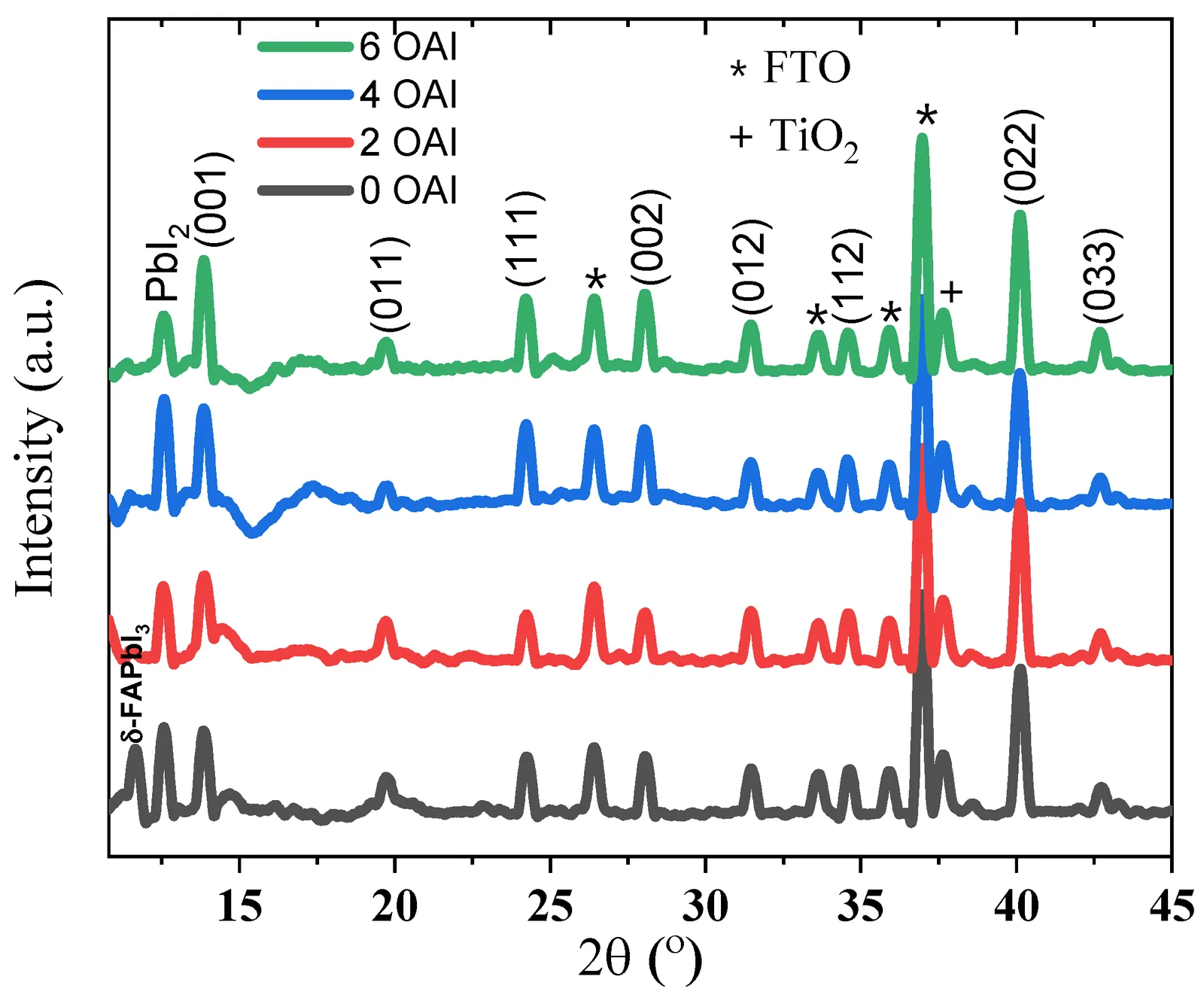
XRD data for the perovskite (MACl)_0.33_FA_0.99_MA_0.01_Pb(I_0.99_Br_0.01_)_3_ film with n-octylammonium iodide concentrations.

Figure 2 displays the XRD patterns of (MACl)_0.33_FA_0.99_MA_0.01_Pb(I_0.99_Br_0.01_)_3_ films passivated with various n-octylammonium iodide (OAI) concentrations that were deposited on glass/FTO/c-TiO_2_/m-TiO_2_. The diffraction peaks of the α-FAPbI_3_ phase appeared at a 2θ of 13.76, 19.57, 24.21, 28.02, 31.34, 34.49, 40.14, and 42.63°, which were associated with the (001), (011), (111), (002), (012), (112), (022), and (033) planes, respectively [46]. A diffraction peak of the δ-FAPbI_3_ phase (unwanted) was detected at 2θ of 11.65° [47] for the perovskite layer without OAI. Using OAI, no δ-FAPbI_3_ signal should be detected in the prepared perovskite films. The (001) diffraction peak of hexagonal PbI_2_ was detected at 12.6° for the perovskite films. Furthermore, there were three additional peaks at 26.36, 33.67, and 35.82°, which represented the FTO layer, and one peak at 37.64°, which can be assigned to the TiO_2_ phase [48]. Besides, the addition of OAI to the system did not affect the peak positions of the α-FAPbI_3_, which indicated the unchanging nature of the lattice constant of the α-FAPbI_3_ phase. On the other hand, the presence of OAI influenced the peaks’ intensities, which indicated a change in the crystallinity of the film.

**Figure 3:**
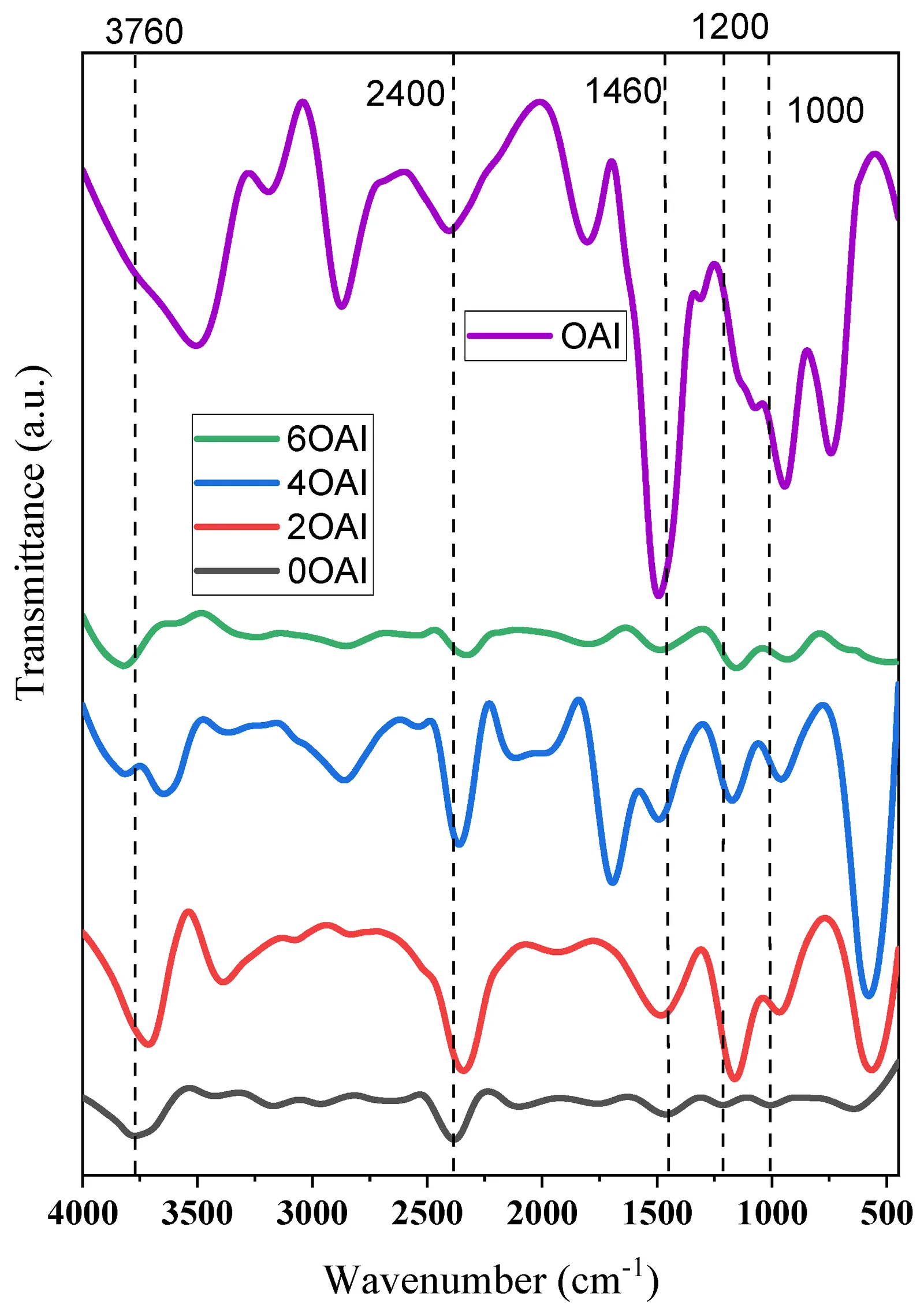
FTIR spectra for the perovskite (MACl)_0.33_FA_0.99_MA_0.01_Pb(I_0.99_Br_0.01_)_3_ film with n-octylammonium iodide concentrations.

Each passivant (2, 4, and 6 mg/mL in isopropanol) was coated on top of perovskite (MACl)_0.33_FA_0.99_MA_0.01_Pb(I_0.99_Br_0.01_)_3_ in order to investigate the potential interactions between the passivants and perovskite. Fourier transform infrared (FTIR) spectroscopy was used to study changes in the chemical bonds (Figure 3). Five distinct changes could be observed in the IR spectra following the OAI treatment. (i) A broad peak centered around 3760 cm^−1^ was produced in all the OAI-treated samples, signaling the structural H-O-H groups’ stretching vibrations [49]. (ii) A strong band appeared around 2400 cm^−1^, which can be assigned to the structural N−H groups. (iii) The N-H rocking bands ranged between 1100 and 1300 cm^−1^ [50]. (iv) In addition, the NH_3_ asymmetric bending mode, which indicates FA^+^, occurred in the 1460–1472 cm^−1^ region [51,52]. (v) The C-N rocking bands, which revealed the perovskite layer, were apparent in the 960–1000 cm^−1^ range [52]. The OAI-treated perovskite film peaks’ blue and red shifts were evidence of the strong symmetrical interactions between the perovskite films and the functional groups of OAI. According to the FTIR data, OAI can passivate the defects of the interfaces and grain boundaries of perovskite films.

**Figure 4:**
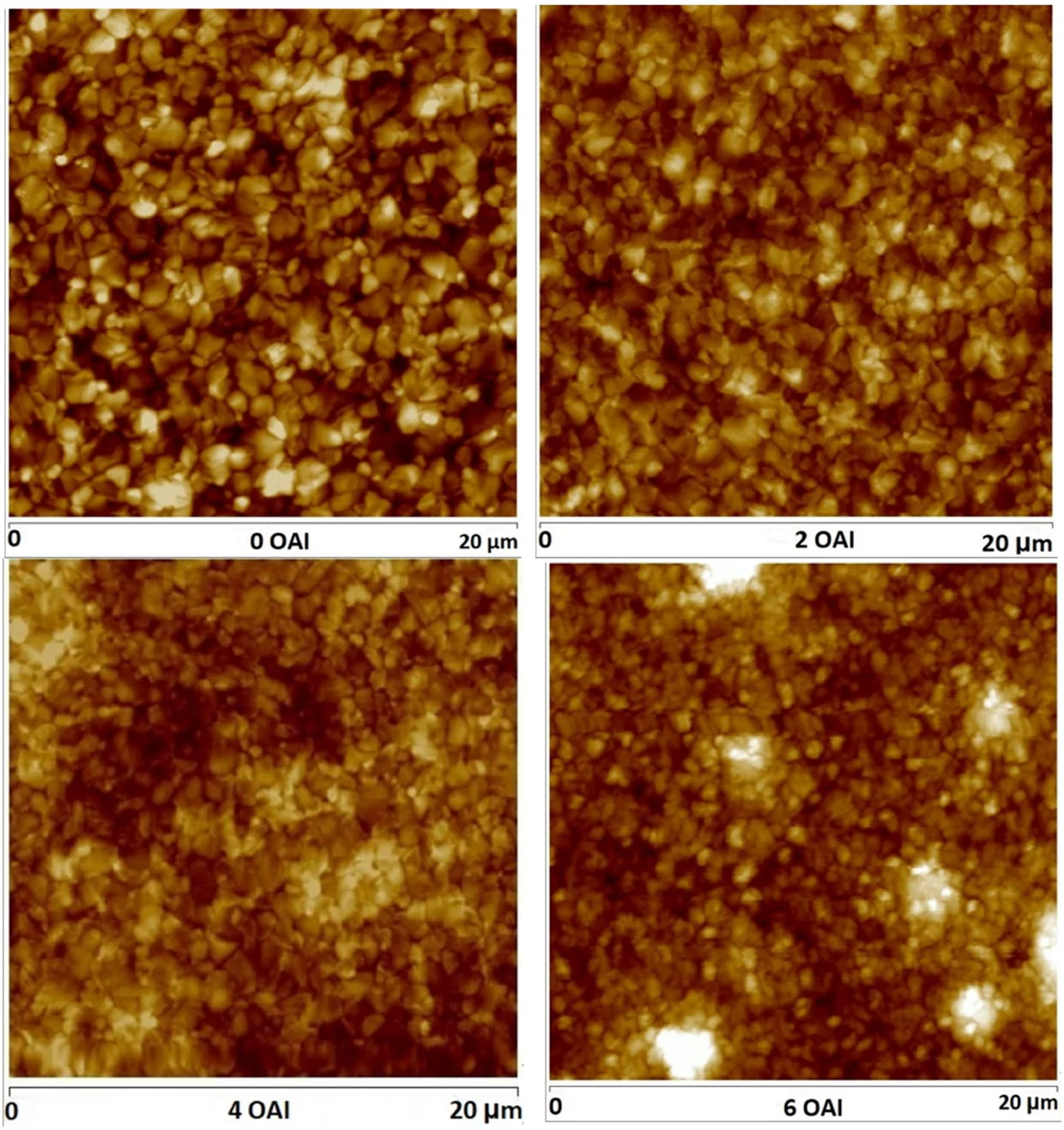
AFM topography images at a scale of 20 μm × 20 μm for the perovskite (MACl)_0.33_FA_0.99_MA_0.01_Pb(I_0.99_Br_0.01_)_3_ film with n-octylammonium iodide concentrations.

Figure 4 displays the results of atomic force microscopy (AFM), which determine how passivation affects the morphology of perovskite films. Figure 4 depicts the AFM micrographs of the samples at a scale of 20 μm × 20 μm. The surface of perovskite film without OAI was rough with an average roughness (Ra) and root-mean-squared roughness (RMS) of about 42.6 and 53.5 nm, respectively. After OAI passivation, there were significant changes in the grain size, grain boundaries, and surface roughness. This showed that the post-treatment passivation had an impact on the perovskite morphology. The surface roughness increased as the OAI concentration increased, as shown in Table 1. Compared to pristine perovskite films, the perovskite films with OAI were smoother with a smaller surface roughness between 32.8 and 36.6 nm. As shown in Table 1, the surface roughness increased with the increase in the concentration of OAI. Nonradiative recombination decreased as the surface roughness decreased. Furthermore, the small surface roughness was favorable for HTM layer deposition and reduced the interface resistance to speed up the charge transfer [53].

**Figure 5:**
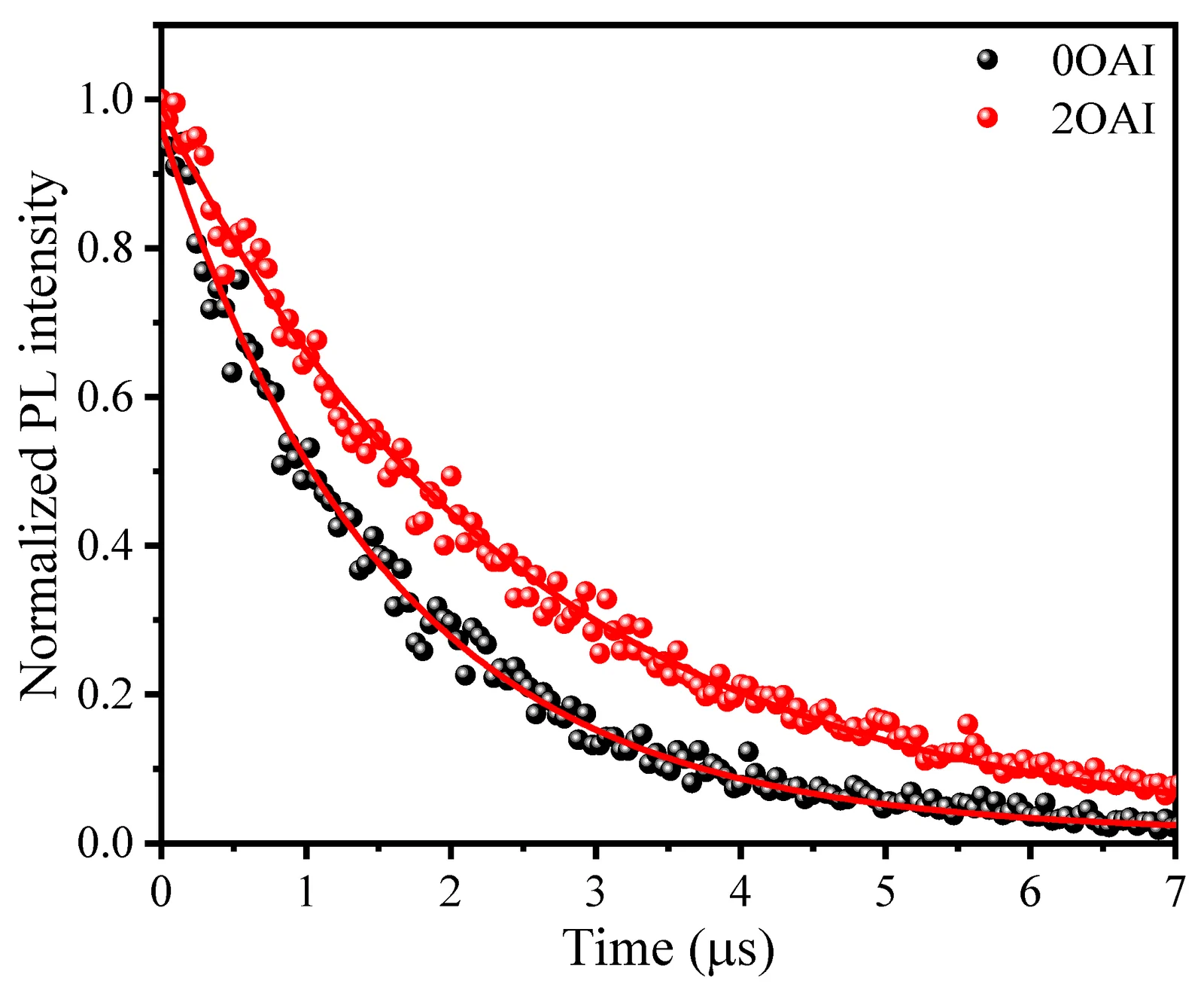
Time-resolved photoluminescence of the perovskite films post-treated with OAI.

To investigate the change in the charge carrier dynamics, we employed time-resolved photoluminescence (TRPL). We measured the TRPL spectra for both the 0OAI and 2OAI films deposited on the glass substrate (Figure 5). The decay time was obtained for both films by fitting the TRPL curves with a mono-exponential function. We observed a significant improvement where the lifetime was more than 60% longer for the 2 OAI in comparison to the 0 OAI films, as shown in Table 2. This indicated a suppression of nonradiative recombination at the surface of the perovskite layer, leading to better interfacial contact between the perovskite and the HTL.

**Figure 6:**
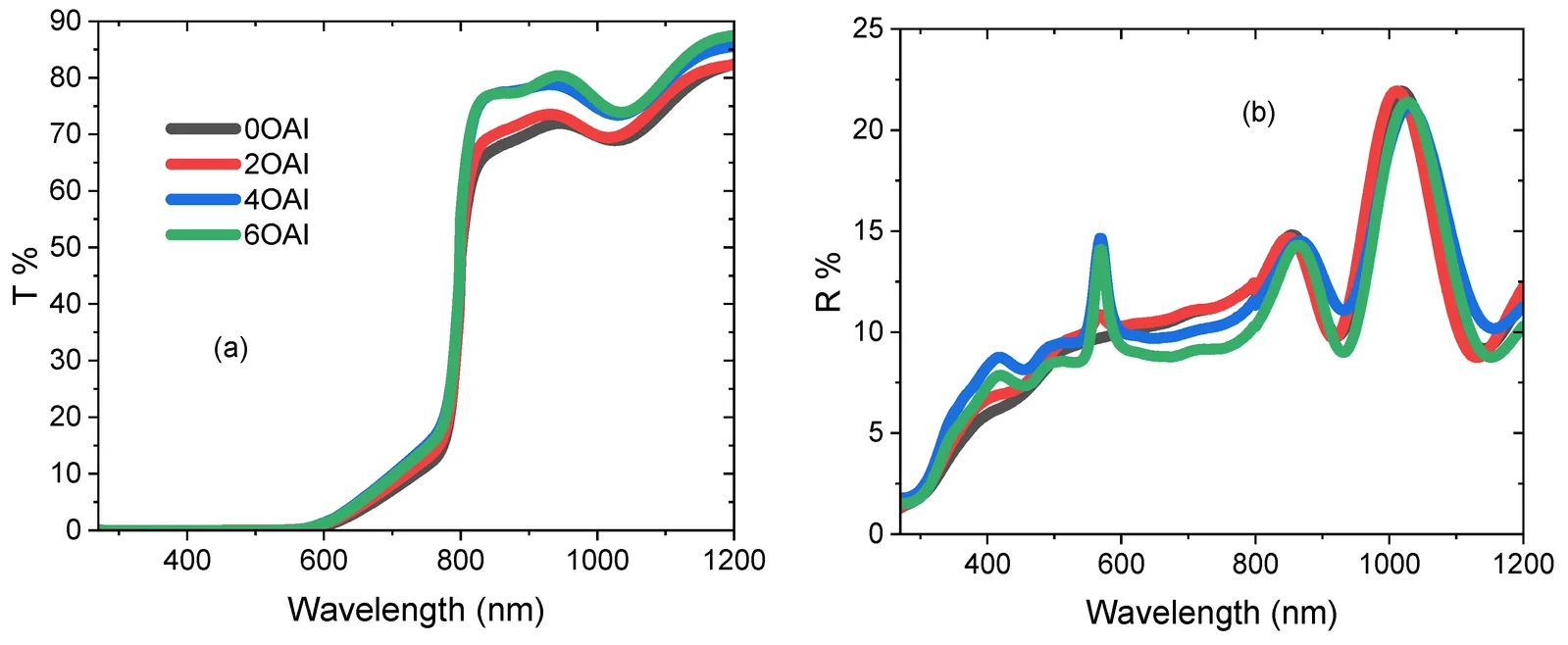
(**a**) The transmittance and (**b**) reflectance spectra for the perovskite (MACl)_0.33_FA_0.99_MA_0.01_Pb(I_0.99_Br_0.01_)_3_ films depend on the wavelength with n-octylammonium iodide concentrations.

The optical performance of hybrid perovskite films is essential for efficient photovoltaic devices. Figure 6 shows the spectrum distribution of transmission T(*λ*) and reflection R(*λ*) of glass/FTO/c-TiO_2_/m-TiO_2_/(MACl)_0.33_FA_0.99_MA_0.01_Pb(I_0.99_Br_0.01_)_3_ films with different concentrations of OAI. In general, the value of T(*λ*) was less than 2% in the region between 190 and 600 nm; however, it gradually increased with increasing *λ* in the visible–near infrared region (600–1200 nm). In the region above *λ* = 800 nm, all films almost became transparent (i.e., Reflectance + Transmittance = 1) due to the limited energy loss as a result of scattering. The inequality (Reflectance + Transmittance < 1) at shorter wavelengths (*λ* < 600 nm) was caused by the presence of absorption. The enhancement of optically homogeneous thin films was indicated by longer-wavelength interference spectra [54]. From the transmittance spectra, films with 4 and 6 OAI had the maximum transmittance. Furthermore, the reflectance gradually increased as the wavelength ranged from 190 to 1200 nm. The interference phenomenon led to some reflectance oscillations at different wavelengths [55]. The reflectance oscillations proved the films’ optical homogeneity [55].

**Figure 7:**
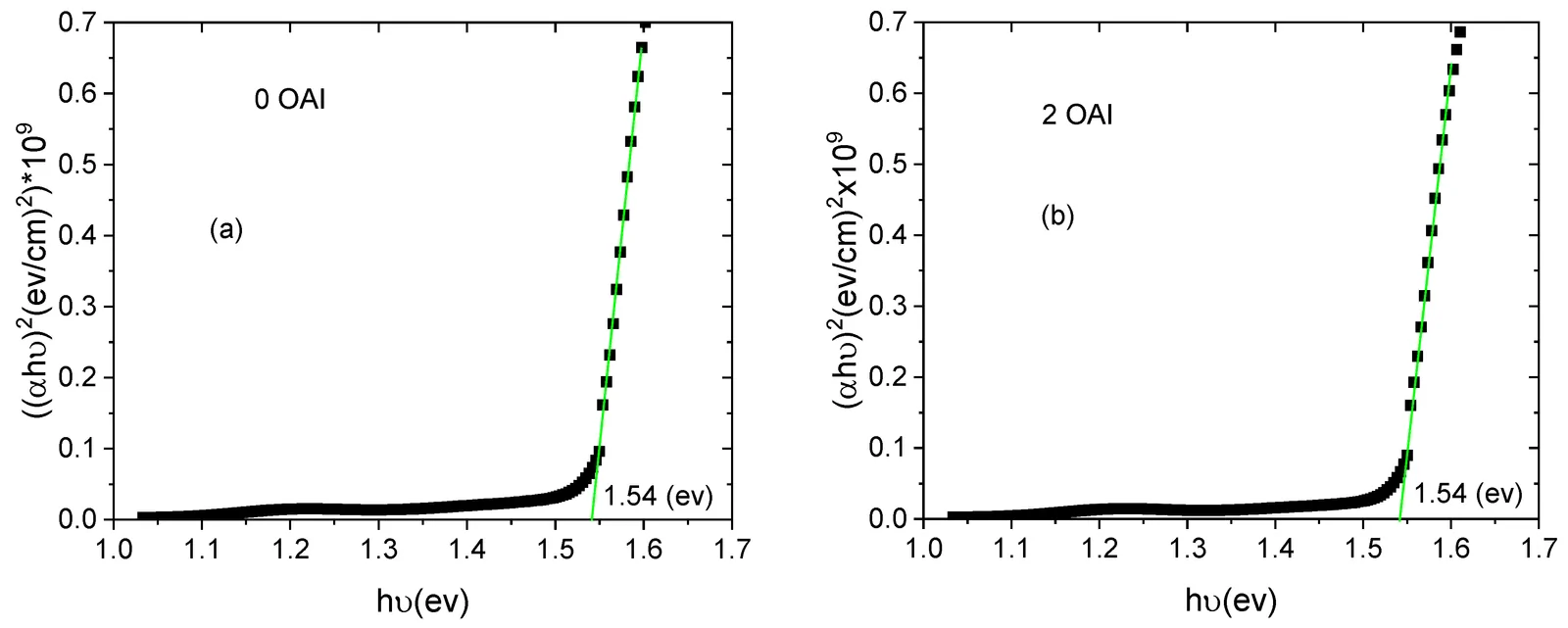

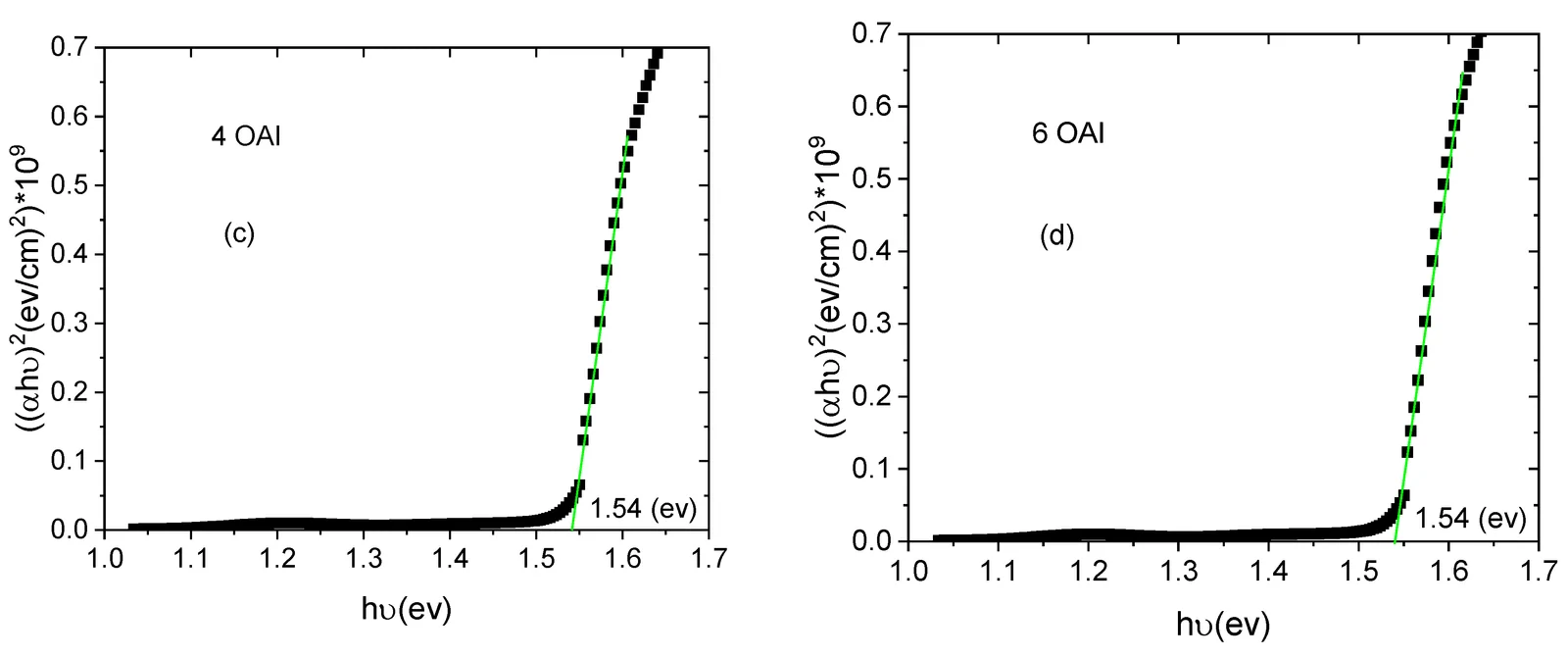
(**a**–**d**) Tauc plot results for the perovskite (MACl)_0.33_FA_0.99_MA_0.01_Pb(I_0.99_Br_0.01_)_3_ films with n-octylammonium iodide concentrations, where the extrapolation of the green line will intercept with the energy band gap.

The electronic transition in semiconductor materials can be illustrated using band theory [56]. An electron in molecules moves from the highest occupied molecular orbital (HOMO) to the lowest unoccupied molecular orbital (LUMO) when electromagnetic radiation is absorbed [56]. Therefore, for a molecular crystal, the HOMO orbital (π-orbital) shares the valence band (VB), while the conduction band (CB) is formed by the combination of the LUMO orbitals ( $\pi^{*}$ -orbitals). The band gap is between VB and CB. The band gap (E_g_) can be computed from the spectral position of the optical absorption edge. The best fitting of the experimental data to Tauc’s equation (αhυ = B(hυ − E_g_)^ɣ^) was obtained when γ = 0.5, so the direct allowed transition was the type of electronic transition [57,58], which is represented in Figure 7 for glass/FTO/c-TiO_2_/m-TiO_2_/(MACl)_0.33_FA_0.99_MA_0.01_Pb(I_0.99_Br_0.01_)_3_ films with a different concentration of OAI. The values of E_g_ for all films were found to be 1.54 eV, which were not impacted by the OAI amount. The unchanging band gap is compatible with the lattice parameters remaining unchanged, as discussed in the XRD part. When alkaline earth metal chloride, BCl_2_ (B = Mg, Ca, Sr, and Ba), was added to the FAPbI_3_ perovskite, a similar result was found [59].

**Figure 8:**
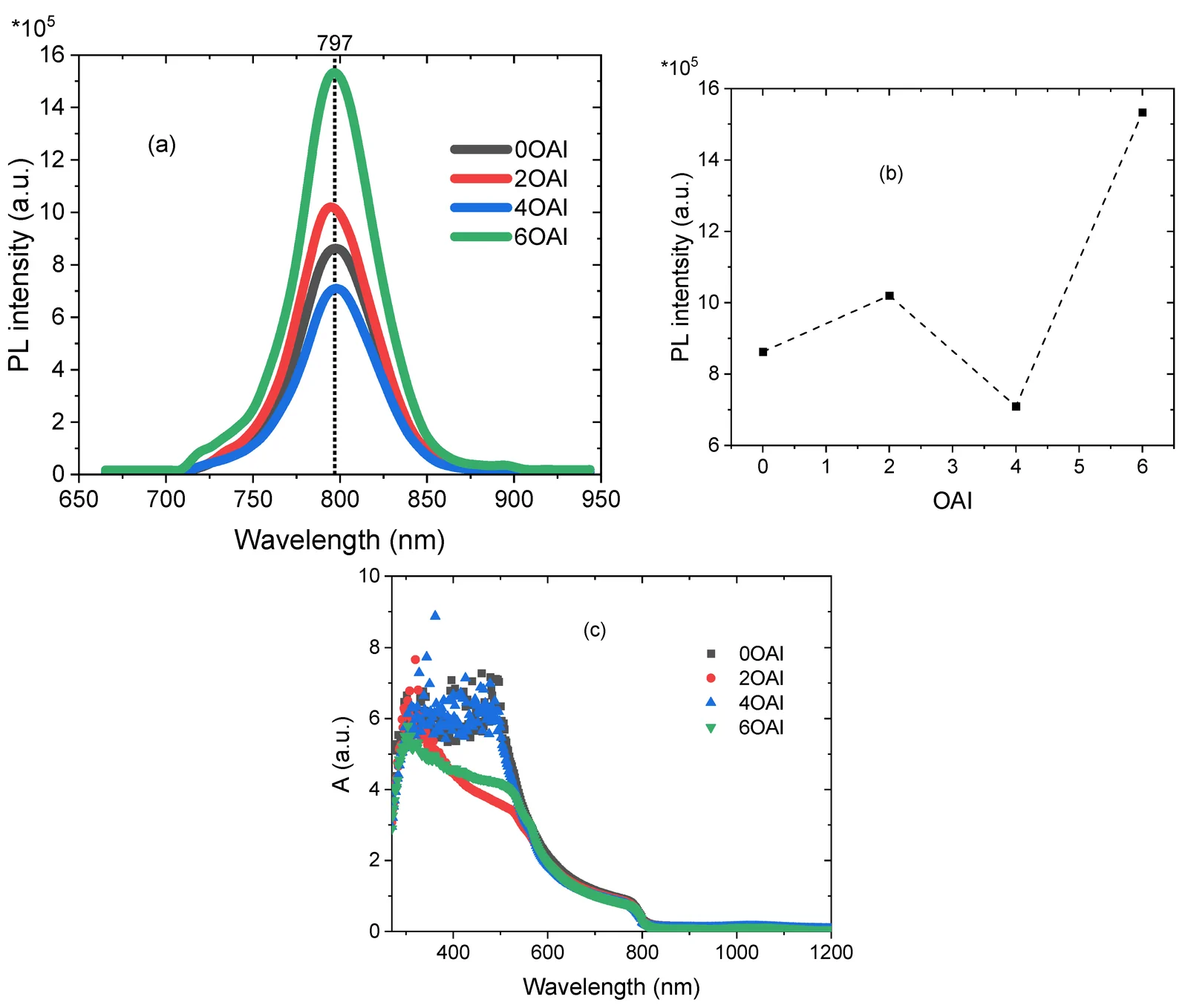
(**a**) PL spectra, (**b**) the changes in the PL intensity, and (**c**) absorption spectra for the perovskite (MACl)_0.33_FA_0.99_MA_0.01_Pb(I_0.99_Br_0.01_)_3_ films with n-octylammonium iodide concentrations.

The photoluminescence (PL) emissions of glass/FTO/c-TiO_2_/m-TiO_2_/(MACl)_0.33_FA_0.99_MA_0.01_Pb(I_0.99_Br_0.01_)_3_ films with and without OAI were also examined. As revealed in Figure 8a, the perovskite steady-state PL graphs showed a typical and high luminescence peak at sites that matched the absorption edges seen in UV–Vis measurements (Figure 8c). All layers of perovskite samples (FTO, c-TiO_2_, m-TiO_2_, and perovskite) were the same for all samples, so the change in PL intensity was affected by the OAI-passivated layer. In contrast to the film without OAI, the films of (MACl)_0.33_FA_0.99_MA_0.01_Pb(I_0.99_Br_0.01_)_3_ with 2OAI and 6OAI had higher PL intensities (Figure 8b), indicating that the OAI additive can greatly reduce trap-mediated and nonradiative recombination in the perovskite layer [60]. The OAI additive affected the crystallinity of those perovskite films (XRD part). The amount of defects created in the entire (MACl)_0.33_FA_0.99_MA_0.01_Pb(I_0.99_Br_0.01_)_3_ film decreased or increased as the crystallinity of the films increased or decreased, and this affected the recombination traps in the perovskite films. Accordingly, the optoelectronic features of perovskite films were improved because the nonradiative recombination pathway was restrained. Conversely, the PL intensity of the (MACl)_0.33_FA_0.99_MA_0.01_Pb(I_0.99_Br_0.01_)_3_ film with 4OAI was reduced because of the increase in the density of microstructural defects [60].

The real component of the complex refractive index is defined as the reflective index (*n*), which is related to the speed of electromagnetic wave propagation throughout thin films and gives the details of electronic polarization. However, the imaginary component is known as the extinction coefficient (*k*), which is linked to the decay of the magnitude of the incident electric field’s oscillations. The following relations were used to determine the values of *n* and *k* for all films [57,58]:

(1) $$n=\frac{1+R}{1-R}+\sqrt{\frac{4R}{{\left(1-R\right)}^{2}}-k^{2}}$$

(2) $$k=\frac{\lambda \alpha }{4\pi }$$

where *α* is the absorption coefficient = 2.303 * bsorbance/thickness of the film.

**Figure 9:**
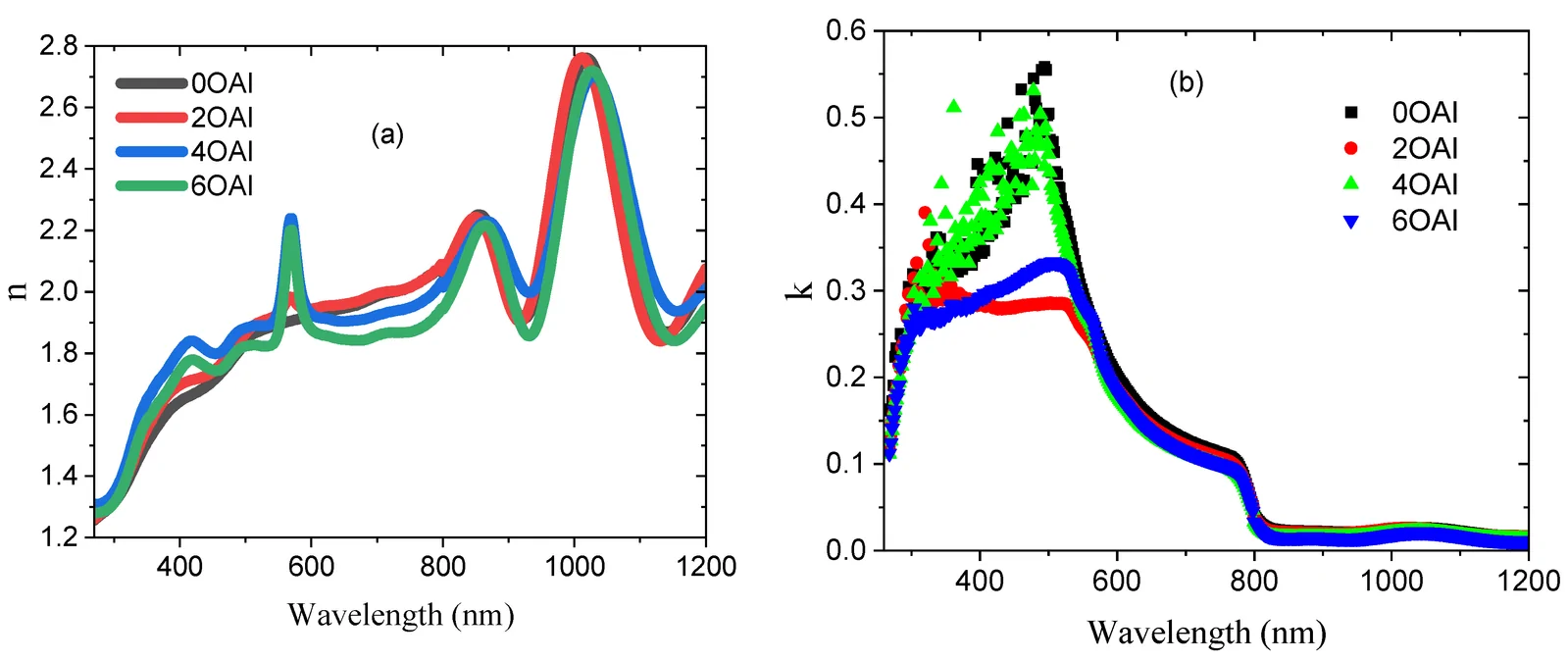
Wavelength variation of the (**a**) refractive index and (**b**) extinction coefficient for the perovskite (MACl)_0.33_FA_0.99_MA_0.01_Pb(I_0.99_Br_0.01_)_3_ films with n-octylammonium iodide concentrations.

The *n* (*λ*) and *k* (*λ*) values for glass/FTO/c-TiO_2_/m-TiO_2_/(MACl)_0.33_FA_0.99_MA_0.01_Pb(I_0.99_Br_0.01_)_3_ films with OAI concentrations are presented in Figure 9. The maximum *n* (*λ*) value was between =1000 and 1100 nm (Figure 9a). Figure 9b shows a maximum value for *k* (*λ*) curves with wavelength values between =300 and 650 nm. The spectrum can be divided into two regions based on the wavelength to study the dispersion of the reflective index. The first region at *λ* < 520 nm (see Figure 9a), in which the *n* values showed an anomalous dispersion, while in the second region *λ* ˃ 520 nm, the *n* values had many peaks (*n* exhibited both normal and anomalous dispersion) [61]. The change in the film’s absorption, density, and polarizability may be the reason for the variation in the *n* and *k* values because of the addition of the OAI layers [62].

Understanding the optical characteristics of semiconductors requires computing the complex dielectric constant (*ε* = *ε_r_* + *iε_i_*). The real component of the dielectric constant (*ε_r_*) provides information about the material’s optical dispersion, whereas the imaginary component of the dielectric constant (*ε_i_*) gives information about the wave’s dissipative rate in the material. The energy loss produced by fast electrons as they pass through the surface and bulk of a material are described by the surface and volume energy loss functions (*SELF*, *VELF*), respectively. The following relations can be used to define the values of *ε_r_*, *ε_i_*, *SELF*, and *VELF* [57,58]:

(3) $$\varepsilon_{r}=n^{2}-k^{2}$$

(4) $$\varepsilon_{i}=2nk$$

(5) $$SELF=\frac{\varepsilon_{i}}{{\left(\varepsilon_{r}+1\right)}^{2}+\varepsilon_{i}^{2}}$$

(6) $$VELF=\frac{\varepsilon_{i}}{\varepsilon_{r}^{2}+\varepsilon_{i}^{2}}$$

**Figure 10:**
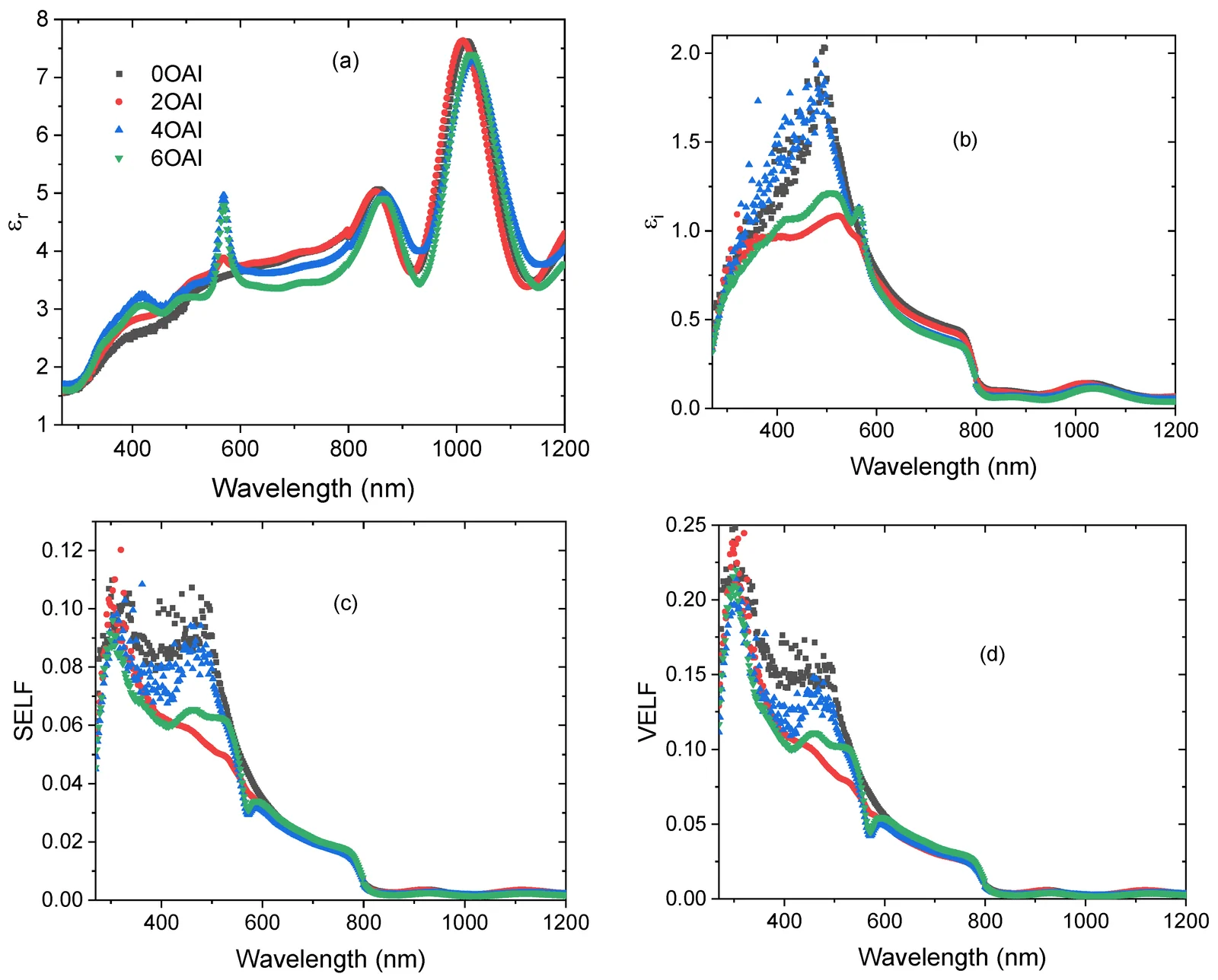
Wavelength variation of the (**a**,**b**) real and imaginary parts of the dielectric constants and (**c**,**d**) surface and volume energy loss function of the perovskite (MACl)_0.33_FA_0.99_MA_0.01_Pb(I_0.99_Br_0.01_)_3_ films with n-octylammonium iodide concentrations.

As revealed in Figure 10a,b, the values of *ε_r_* were higher than the values of *ε_i_*. Furthermore, as the wavelength increased, the values of *ε_r_* increased slightly. Furthermore, the values of *ε_i_* increased with the wavelength up to 400–500 nm, then decreased with the wavelength, remaining stable beyond 800 nm. The addition of OAI affected the dielectric constants, where at the lower wavelength range, *ε_r_* enhanced, while *ε_i_* reduced as the system contained OAI. The charge displacement and the polarization in crystal affected the dielectric constants. The changes in the values of *ε_r_* and *ε_i_* at low wavelengths were assigned to the strong absorption bands.

Figure 10c,d show both *SELF* and *VELF* as functions of wavelength. It was evident that the free charge carriers lost about the same amount of energy when passing through the surface and bulk material. The values of *SELF* were less than the values of *VELF* for the incident photons due to the charge carriers traveling a long distance through the bulk material, resulting in more collisions with the charges within the material [63]. The *SELF* and *VELF* values of all films in the wavelength ranging up to 600 nm changed dramatically depending on the amount of OAI. After that, the *SELF* and *VELF* values of all films were almost unchanged.

The optical conductivity (*σ_opt_*) is an optical characteristic that provides details about the material’s electronic state. Furthermore, it represents the electrical conductivity of the material, which is based on free charge carriers that are produced via the electric field linked to the incident light. The following formula can be used to determine *σ_opt_* [57,58]:

(7) $$\sigma_{opt}=\frac{\alpha nc}{4\pi }$$

where *n*, *α*, and *c* are the refractive index, absorption coefficient of the material, and the velocity of light, respectively.

**Figure 11:**
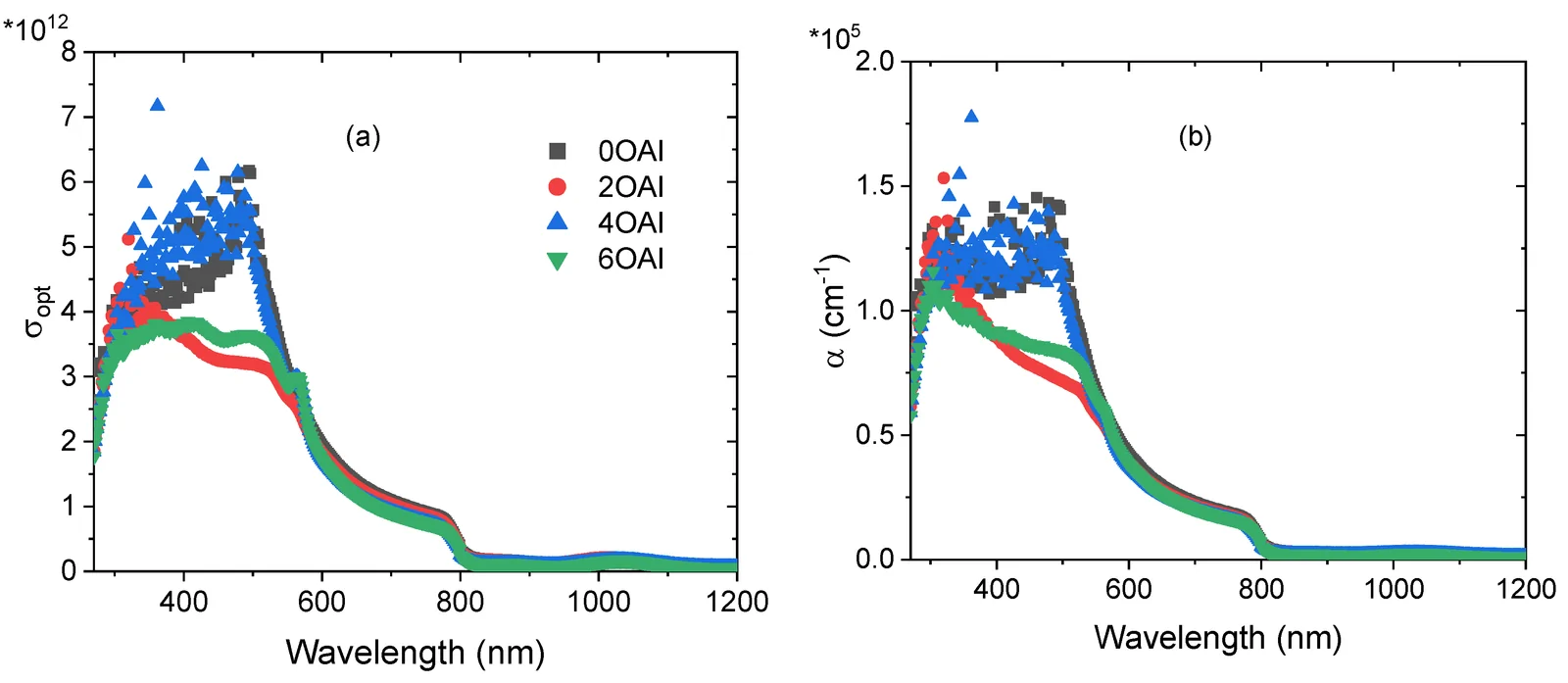
Changes of the (**a**) optical conductivity and (**b**) absorption coefficient with the wavelength of the perovskite (MACl)_0.33_FA_0.99_MA_0.01_Pb(I_0.99_Br_0.01_)_3_ films with n-octylammonium iodide concentrations.

The variation in the wavelength for glass/FTO/c-TiO_2_/m-TiO_2_/(MACl)_0.33_FA_0.99_MA_0.01_Pb(I_0.99_Br_0.01_)_3_ films with different concentrations of OAI are displayed in Figure 11a. As depicted in Figure 11a, the values of *σ_opt_* demonstrated a linear behavior that was symmetric with respect to both *α* and *n* based on Equation (7) (Figure 11b). The graph shows the highest value in the wavelength range between *λ* = 300 and 500 nm, due to the strong absorption through thin films in this region [64]. Beyond this range, *σ_opt_* reduced with increasing wavelength.

The interaction between the electromagnetic field of the incident light and an inherent charge in the material caused a change in its phase, frequency, amplitude, or polarization. The study of these interactions is known as nonlinear optics (NLO). The NLO parameters of materials have significance in their application in many application fields, such as medicine, telecommunications technology, data and imaging, and sensors [65]. According to Miller’s formula, which is dependent on the linear optical susceptibility (*χ*^(1)^), the third-order non-linear optical susceptibility (*χ*^(3)^) and the non-linear refractive index (*n*_2_) can be obtained [57,58]:

(8) $$\chi^{\left(1\right)}=\frac{1}{4\pi }\left(n^{2}-1\right)$$

(9) $$\chi^{\left(3\right)}=1.7*10^{-10}*{\left(\chi^{\left(1\right)}\right)}^{4}=1.7*10^{-10}*{\left(\frac{n^{2}-1}{4\pi }\right)}^{4}$$

(10) $$n_{2}=\frac{12\pi }{n}*\chi^{\left(3\right)}$$

**Figure 12:**
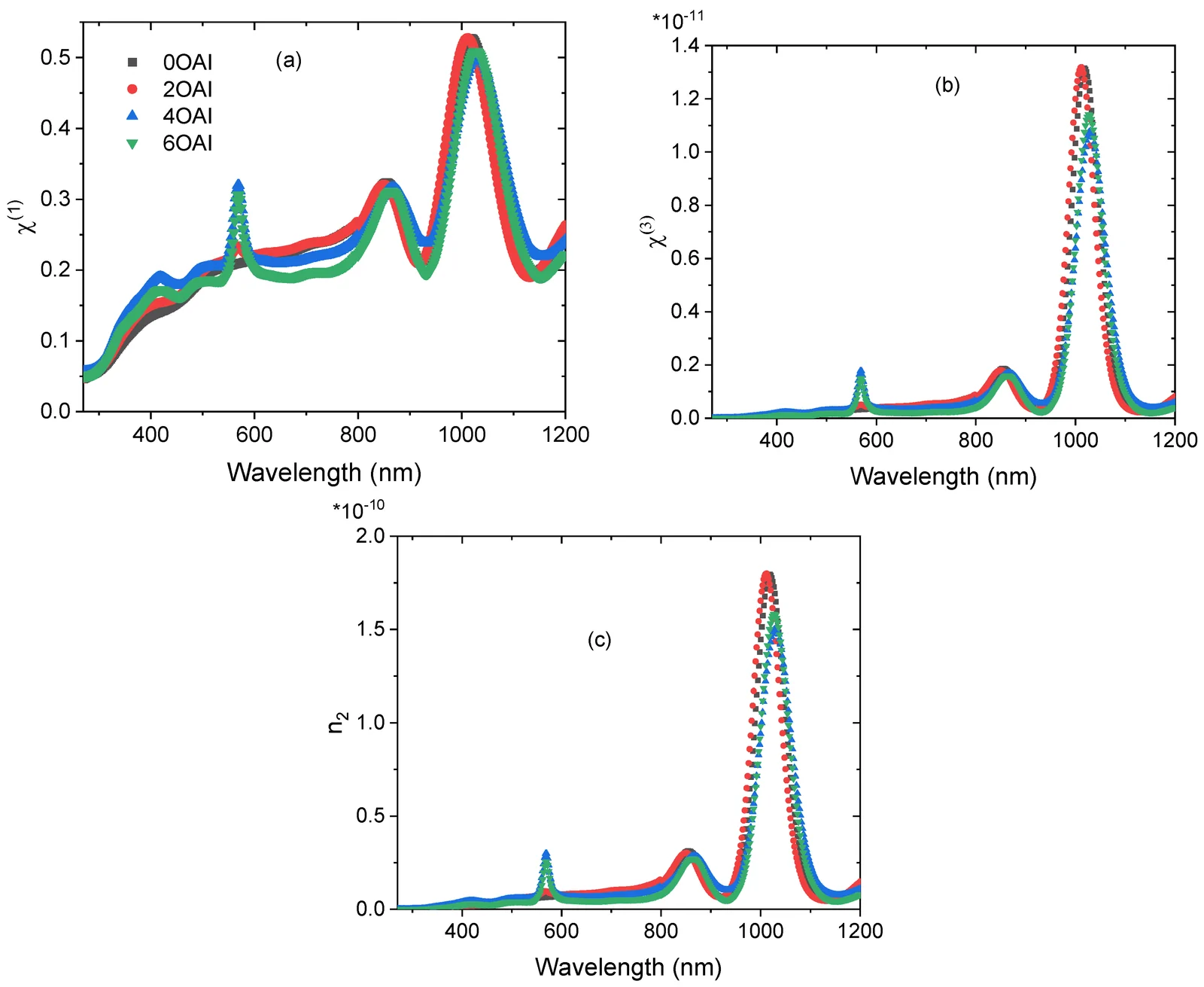
Variation of the (**a**) linear optical susceptibility, (**b**) third-order non-linear optical susceptibility, and (**c**) non-linear refractive index against wavelength for the perovskite (MACl)_0.33_FA_0.99_MA_0.01_Pb(I_0.99_Br_0.01_)_3_ films with n-octylammonium iodide concentrations.

The modifications of the *χ*^(1)^, *χ*^(3)^, and *n*_2_ values for glass/FTO/c-TiO_2_/m-TiO_2_/(MACl)_0.33_FA_0.99_MA_0.01_Pb(I_0.99_Br_0.01_)_3_ films with OAI concentrations against the wavelength are shown in Figure 12a–c. The NLO parameters changed in similarity, as seen in Figure 12a–c, where the *χ*^(1)^, *χ*^(3)^, and n_2_ curves display three peaks around 570, 860, and 1020 nm. The NLO parameters reached their greatest values in the IR region. The NLO parameters for films contained 4 and 6 OAI, which were enhanced at 570 nm, as compared with the other films.

**Figure 13:**
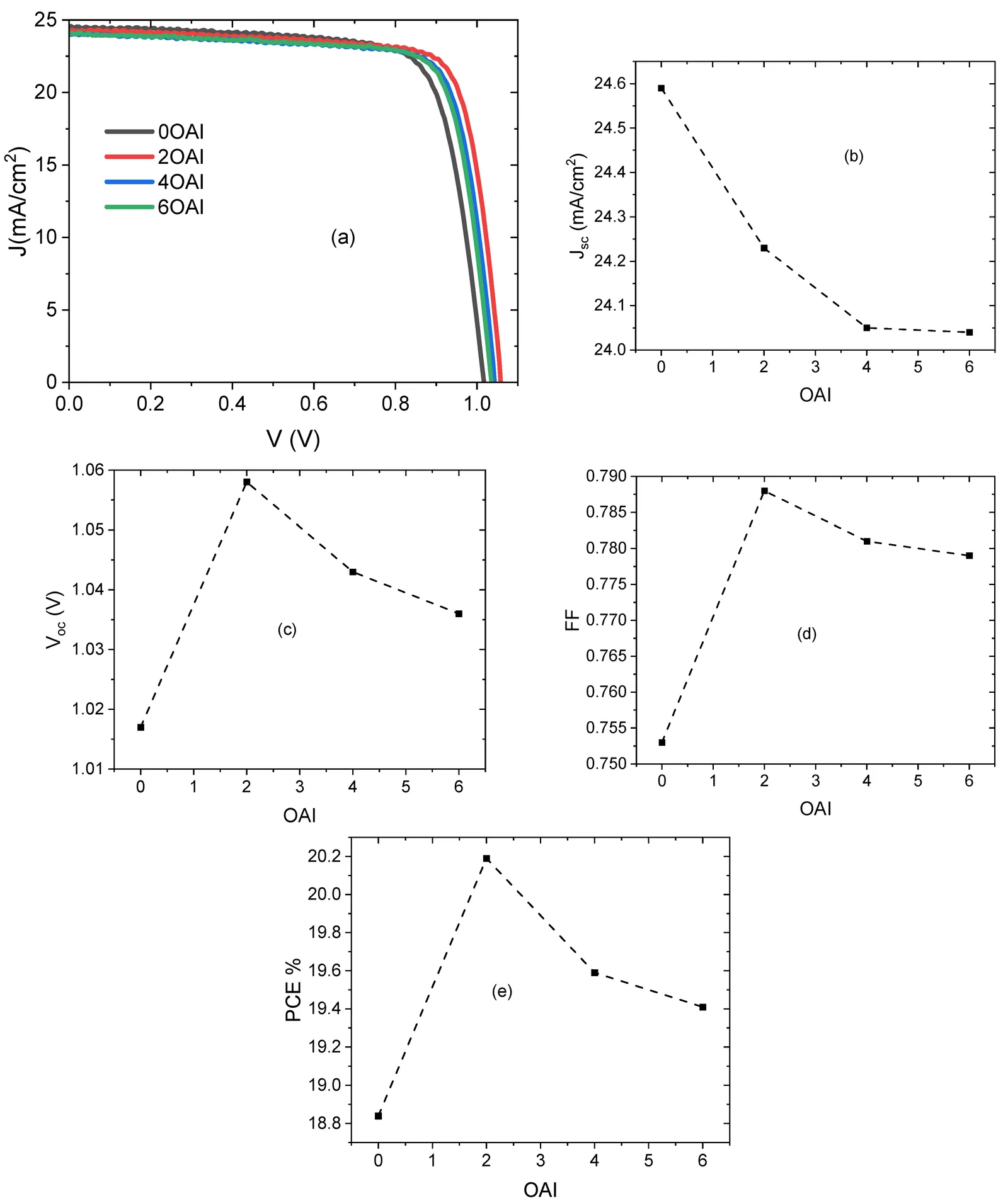
(**a**) Current density−voltage (J–V) curves of the PSCs with n-octylammonium iodide concentrations, change of the (**b**) current density, (**c**) open-circuit voltage, (**d**) fill factor, and (**e**) power conversion efficiency with n-octylammonium iodide concentrations.

The PSCs were fabricated using (MACl)_0.33_FA_0.99_MA_0.01_Pb(I_0.99_Br_0.01_)_3_ perovskite films with OAI concentrations to study the impact of OAI on the photovoltaic parameters. Figure 13a displays the photocurrent density–voltage (J–V) curves for the PSCs, and Table 3 lists the obtained parameters. As revealed from Figure 13a–e and Table 3, the changing concentration of OAI affected the overall PSC devices’ performance. The fabricated PSCs without OAI passivation had a power conversion efficiency (PCE), open-circuit voltage (V_OC_), short-circuit current density (J_SC_), and fill factor (FF) of 18.8%, 1.02 V, 24.6 mAcm^−2^, and 75%, respectively. The efficiency of the fabricated PSCs was impacted by the OAI passivation layer. The V_OC_, FF, and PCE first increased as the amount of OAI became 2 mg, then reduced with more additives of OAI. The value of J_SC_ decreased as the amount of OAI increased. The maximum PCE value of 20.2% was obtained for the passivation layer of a 2 mg OAI concertation. The increase in the PCE can be attributed to the improved V_OC_ (1.06 V) and FF (79%) and the reduction in the J_SC_ (24.2 mAcm^−2^) value after OAI treatment. For the OAI-passivated PSCs, the V_OC_ increased due to a decrease in nonradiative recombination [15,66], while the reduction in J_SC_ indicated the enhancement of charge collection [15]. As shown in Figure 13d, the FF decreased slightly as the amount of OAI additives increased. Furthermore, additional passivation solutions, such as PMMA:PCBM, increased the voltage while decreasing the FF [67,68]. In summary, the OAI-passivated layer enhanced the performance parameters of the PSCs due to the decrease in the recombination trap states and improved charge collection [15].

## 4. Conclusions

The perovskite’s crystal structure was unaffected by the addition of OAI. The addition of OAI did not change the band gap of the films. A typical and high peak luminescence can be seen on all films’ steady-state PL graphs. The PL emission intensity of the film prepared with 6 mg OAI indicated nonradiative carrier recombination. The refractive index values showed both anomalous and/or normal dispersion depending on the wavelength range. As a result of the changes in the optical conductivity and the dielectric constant values caused by the addition of the OAI passivation layer, they may be suitable for use in a variety of solar cell applications. The use of the passivated films in many nonlinear devices is recommended because of the changes in the values of the NLO parameters. As the amount of OAI additive increased, the J_SC_ slightly decreased for all devices, whereas the PCE, V_OC_, and FF were enhanced. The maximum PCE was obtained when the passivation layer amount reached 2 mg of OAI. The decrease in the recombination trap states and the improved charge collection of the OAI-passivated cell were the reasons for the enhanced photovoltaic parameters.

## Figures and Tables

**Table 1:** R_a_ and RMS values determined by AFM for the perovskite (MACl)_0.33_FA_0.99_MA_0.01_Pb(I_0.99_Br_0.01_)_3_ film with n-octylammonium iodide concentrations.

Perovskite Films	R_a_ (nm)	RMS (nm)
(MACl)_0.33_FA_0.99_MA_0.01_Pb(I_0.99_Br_0.01_)_3_	42.6	53.5
(MACl)_0.33_FA_0.99_MA_0.01_Pb(I_0.99_Br_0.01_)_3_/2OAI	32.8	40.6
(MACl)_0.33_FA_0.99_MA_0.01_Pb(I_0.99_Br_0.01_)_3_/4OAI	34	42.6
(MACl)_0.33_FA_0.99_MA_0.01_Pb(I_0.99_Br_0.01_)_3_/6OAI	36.6	51.2

**Table 2:** TRPL lifetime determined by mono-exponential fitting control and target films deposited on glass.

	t_1_ (ns)
0 OAI	1561.20 ± 42.63
2 OAI	2462.52 ± 26.67

**Table 3:** Photovoltaics data of PSCs with n-octylammonium iodide concentrations.

Perovskite Solar Cells	J_SC_ (mA/cm^2^)	V_OC_ (V)	FF	PCE (%)
(MACl)_0.33_FA_0.99_MA_0.01_Pb(I_0.99_Br_0.01_)_3_	24.6	1.02	0.75	18.8
(MACl)_0.33_FA_0.99_MA_0.01_Pb(I_0.99_Br_0.01_)_3_/2OAI	24.2	1.06	0.79	20.2
(MACl)_0.33_FA_0.99_MA_0.01_Pb(I_0.99_Br_0.01_)_3_/4OAI	24.1	1.04	0.78	19.6
(MACl)_0.33_FA_0.99_MA_0.01_Pb(I_0.99_Br_0.01_)_3_/6OAI	24.0	1.04	0.78	19.4

## Data Availability

Data will be made available upon request.

## References

[1] Tavakoli M.M., Tress W., Milić J.V., Kubicki D., Emsley L., Grätzel M. (2018). Addition of adamantylammonium iodide to hole transport layers enables highly efficient and electroluminescent perovskite solar cells. Energy Environ. Sci..

[2] Jeon N.J., Na H., Jung E.H., Yang T.Y., Lee Y.G., Kim G., Shin H.W., Seok S.I., Lee J., Seo J. (2018). A fluorene-terminated hole-transporting material for highly efficient and stable perovskite solar cells. Nat. Energy.

[3] Xing G., Mathews N., Sun S., Lim S.S., Lam Y.M., Grätzel M., Mhaisalkar S., Sum T.C. (2013). Long-range balanced electron-and hole-transport lengths in organic-inorganic CH_3_NH_3_PbI_3_. Science.

[4] Tavakoli M.M., Zakeeruddin S.M., Grätzel M., Fan Z. (2018). Large-grain tin-rich perovskite films for efficient solar cells via metal alloying technique. Adv. Mater..

[5] Liu S., Guan Y., Sheng Y., Hu Y., Rong Y., Mei A., Han H. (2020). A review on additives for halide perovskite solar cells. Adv. Energy Mater..

[6] Green M.A., Ho-Baillie A., Snaith H.J. (2014). The emergence of perovskite solar cells. Nat. Photonics.

[7] Kojima A., Teshima K., Shirai Y., Miyasaka T. (2009). Organometal halide perovskites as visible-light sensitizers for photovoltaic cells. J. Am. Chem. Soc..

[8] National Renewable Energy Laboratory (NREL) Best Research-Cell Efficiency Chart. https://www.nrel.gov/pv/cell-efficiency.html.

[9] Luo D., Yang W., Wang Z., Sadhanala A., Hu Q., Su R., Shivanna R., Trindade G.F., Watts J.F., Xu Z. (2018). Enhanced photovoltage for inverted planar heterojunction perovskite solar cells. Science.

[10] Sha W.E., Ren X., Chen L., Choy W.C. (2015). The efficiency limit of CH3NH3PbI3 perovskite solar cells. Appl. Phys. Lett..

[11] Sherkar T.S., Momblona C., Gil-Escrig L., Avila J., Sessolo M., Bolink H.J., Koster L.J.A. (2017). Recombination in perovskite solar cells: Significance of grain boundaries, interface traps, and defect ions. ACS Energy Lett..

[12] Wetzelaer G.J.A., Scheepers M., Sempere A.M., Momblona C., Ávila J., Bolink H.J. (2015). Trap-assisted non-radiative recombination in organic–inorganic perovskite solar cells. Adv. Mater..

[13] Luo D., Su R., Zhang W., Gong Q., Zhu R. (2020). Minimizing non-radiative recombination losses in perovskite solar cells. Nat. Rev. Mater..

[14] Rajagopal A., Yao K., Jen A.K.Y. (2018). Toward perovskite solar cell commercialization: A perspective and research roadmap based on interfacial engineering. Adv. Mater..

[15] Mozaffari N., Duong T., Shehata M.M., Bui A.D., Pham H.T., Yin Y., Mayon Y.O., Zheng J., Mahmud M.A., Tabi G.D. (2022). Above 23% efficiency by binary surface passivation of perovskite solar cells using guanidinium and octylammonium spacer cations. Sol. RRL.

[16] Hwang I., Jeong I., Lee J., Ko M.J., Yong K. (2015). Enhancing stability of perovskite solar cells to moisture by the facile hydrophobic passivation. ACS Appl. Mater. Interfaces.

[17] Zhang X., Wu G., Fu W., Qin M., Yang W., Yan J., Zhang Z., Lu X., Chen H. (2018). Orientation regulation of phenylethylammonium cation based 2D perovskite solar cell with efficiency higher than 11%. Adv. Energy Mater..

[18] Stoumpos C.C., Soe C.M.M., Tsai H., Nie W., Blancon J.C., Cao D.H., Liu F., Traoré B., Katan C., Even J. (2017). High members of the 2D Ruddlesden-Popper halide perovskites: Synthesis, optical properties, and solar cells of (CH_3_(CH_2_)_3_NH_3_)_2_(CH_3_NH_3_)_4_Pb_5_I_16_. Chem.

[19] Cao D.H., Stoumpos C.C., Farha O.K., Hupp J.T., Kanatzidis M.G. (2015). 2D homologous perovskites as light-absorbing materials for solar cell applications. J. Am. Chem. Soc..

[20] Cohen B.E., Wierzbowska M., Etgar L. (2017). High efficiency and high open circuit voltage in quasi 2D perovskite based solar cells. Adv. Funct. Mater..

[21] Jiang Q., Zhao Y., Zhang X., Yang X., Chen Y., Chu Z., Ye Q., Li X., Yin Z., You J. (2019). Surface passivation of perovskite film for efficient solar cells. Nat. Photonics.

[22] Grancini G., Roldán-Carmona C., Zimmermann I., Mosconi E., Lee X., Martineau D., Narbey S., Oswald F., De Angelis F., Graetzel M. (2017). One-Year stable perovskite solar cells by 2D/3D interface engineering. Nat. Commun..

[23] Lin D., Zhang T., Wang J., Long M., Xie F., Chen J., Wu B., Shi T., Yan K., Xie W. (2019). Stable and scalable 3D-2D planar heterojunction perovskite solar cells via vapor deposition. Nano Energy.

[24] Huang W., Sadhu S., Sapkota P., Ptasinska S. (2018). In situ identification of cation-exchange-induced reversible transformations of 3D and 2D perovskites. Chem. Commun..

[25] Koh T.M., Shanmugam V., Guo X., Lim S.S., Filonik O., Herzig E.M., Müller-Buschbaum P., Swamy V., Chien S.T., Mhaisalkar S.G. (2018). Enhancing moisture tolerance in efficient hybrid 3D/2D perovskite photovoltaics. J. Mater. Chem. A.

[26] Jung E.H., Jeon N.J., Park E.Y., Moon C.S., Shin T.J., Yang T.Y., Noh J.H., Seo J. (2019). Efficient, stable and scalable perovskite solar cells using poly (3-hexylthiophene). Nature.

[27] Smith I.C., Hoke E.T., Solis-Ibarra D., McGehee M.D., Karunadasa H.I. (2014). A layered hybrid perovskite solar-cell absorber with enhanced moisture stability. Angew. Chem. Int. Ed..

[28] Yoo J.J., Wieghold S., Sponseller M.C., Chua M.R., Bertram S.N., Hartono N.T.P., Tresback J.S., Hansen E.C., Correa-Baena J.P., Bulović V. (2019). An interface stabilized perovskite solar cell with high stabilized efficiency and low voltage loss. Energy Environ. Sci..

[29] Cho K.T., Grancini G., Lee Y., Oveisi E., Ryu J., Almora O., Tschumi M., Schouwink P.A., Seo G., Heo S. (2018). Selective growth of layered perovskites for stable and efficient photovoltaics. Energy Environ. Sci..

[30] Lin Y., Bai Y., Fang Y., Chen Z., Yang S., Zheng X., Tang S., Liu Y., Zhao J., Huang J. (2018). Enhanced thermal stability in perovskite solar cells by assembling 2D/3D stacking structures. J. Phys. Chem. Lett..

[31] Chen P., Bai Y., Wang S., Lyu M., Yun J.H., Wang L. (2018). In situ growth of 2D perovskite capping layer for stable and efficient perovskite solar cells. Adv. Funct. Mater..

[32] Cho Y., Soufiani A.M., Yun J.S., Kim J., Lee D.S., Seidel J., Deng X., Green M.A., Huang S., Ho-Baillie A.W. (2018). Mixed 3D–2D passivation treatment for mixed-cation lead mixed-halide perovskite solar cells for higher efficiency and better stability. Adv. Energy Mater..

[33] Yoo H.S., Park N.G. (2018). Post-treatment of perovskite film with phenylalkylammonium iodide for hysteresis-less perovskite solar cells. Sol. Energy Mater. Sol. Cells.

[34] Luo W., Wu C., Wang D., Zhang Y., Zhang Z., Qi X., Zhu N., Guo X., Qu B., Xiao L. (2019). Efficient and stable perovskite solar cell with high open-circuit voltage by dimensional interface modification. ACS Appl. Mater. Interfaces.

[35] Li N., Zhu Z., Dong Q., Li J., Yang Z., Chueh C.C., Jen A.K.Y., Wang L. (2017). Enhanced Moisture Stability of Cesium-Containing Compositional Perovskites by a Feasible Interfacial Engineering. Adv. Mater. Interfaces.

[36] Zhang F., Kim D.H., Zhu K. (2018). 3D/2D multidimensional perovskites: Balance of high performance and stability for perovskite solar cells. Curr. Opin. Electrochem..

[37] Gharibzadeh S., Abdollahi Nejand B., Jakoby M., Abzieher T., Hauschild D., Moghadamzadeh S., Schwenzer J.A., Brenner P., Schmager R., Haghighirad A.A. (2019). Record open-circuit voltage wide-bandgap perovskite solar cells utilizing 2D/3D perovskite heterostructure. Adv. Energy Mater..

[38] Jang Y.W., Lee S., Yeom K.M., Jeong K., Choi K., Choi M., Noh J.H. (2021). Intact 2D/3D halide junction perovskite solar cells via solid-phase in-plane growth. Nat. Energy.

[39] Heo J.H., Im S.H., Noh J.H., Mandal T.N., Lim C.S., Chang J.A., Lee Y.H., Kim H.J., Sarkar A., Nazeeruddin M.K. (2013). Efficient inorganic–organic hybrid heterojunction solar cells containing perovskite compound and polymeric hole conductors. Nat. Photonics.

[40] Jeon N.J., Noh J.H., Kim Y.C., Yang W.S., Ryu S., Seok S.I. (2014). Solvent engineering for high-performance inorganic–organic hybrid perovskite solar cells. Nat. Mater..

[41] Osterloh F.E. (2014). Maximum theoretical efficiency limit of photovoltaic devices: Effect of band structure on excited state entropy. J. Phys. Chem. Lett..

[42] Umari P., Mosconi E., De Angelis F. (2014). Relativistic GW calculations on CH_3_NH_3_PbI_3_ and CH_3_NH_3_SnI_3_ perovskites for solar cell applications. Sci. Rep..

[43] Du M.H. (2014). Efficient carrier transport in halide perovskites: Theoretical perspectives. J. Mater. Chem. A.

[44] El-naggar A.M., Osman M.M., Aldhafiri A.M., Albassam A.A., Kamal A.M., Mohamed M.B. (2023). Effect of Li-salt additives on the optical features and solar cell performance of Cs_0.05_FA_0.85_MA_0.10_Pb(I_0.90_Br_0.10_)_3_ perovskite solar cells. Opt. Mater..

[45] El-naggar A.M., Osman M.M., Alanazi A.Q., Aldhafiri A.M., Albassam A.A., Kamal A.M., Mohamed M.B. (2023). Comparative study of the optical, structural, and solar cell performance of (MAPbBr_3_)_x_(FAPbI_3_)_1-x_(MACl)_0.33_ mixed perovskite solar cells: With and without the passivation layer. Opt. Mater..

[46] Murugadoss G., Thangamuthu R., Kumar M.R. (2018). Formamidinium lead iodide perovskite: Structure, shape and optical tuning via hydrothermal method. Mater. Lett..

[47] Wang J., Liu L., Chen S., Ran G., Zhang W., Zhao M., Zhao C., Lu F., Jiu T., Li Y. (2022). Growth of 2D passivation layer in FAPbI3 perovskite solar cells for high open-circuit voltage. Nano Today.

[48] Khan M.I., Bhatti K.A., Qindeel R., Althobaiti H.S., Alonizan N. (2017). Structural, electrical and optical properties of multilayer TiO_2_ thin films deposited by sol–gel spin coating. Results Phys..

[49] Slaný M., Jankovič Ľ., Madejová J. (2019). Structural characterization of organo-montmorillonites prepared from a series of primary alkylamines salts: Mid-IR and near-IR study. Appl. Clay Sci..

[50] Wang R.T., Xu A.F., Li W., Li Y., Xu G. (2021). Moisture-stable FAPbI3 perovskite achieved by atomic structure negotiation. J. Phys. Chem. Lett..

[51] Hills-Kimball K., Nagaoka Y., Cao C., Chaykovsky E., Chen O. (2017). Synthesis of formamidinium lead halide perovskite nanocrystals through solid–liquid–solid cation exchange. J. Mater. Chem. C.

[52] Mahmud M.A., Pham H.T., Duong T., Yin Y., Peng J., Wu Y., Liang W., Li L., Kumar A., Shen H. (2021). Combined Bulk and Surface Passivation in Dimensionally Engineered 2D-3D Perovskite Films via Chlorine Diffusion. Adv. Funct. Mater..

[53] Carrillo J., Guerrero A., Rahimnejad S., Almora O., Zarazua I., Mas-Marza E., Bisquert J., Garcia-Belmonte G. (2016). Ionic reactivity at contacts and aging of methylammonium lead triiodide perovskite solar cells. Adv. Energy Mater..

[54] Hernández-Granados A., Corpus-Mendoza A.N., Moreno-Romero P.M., Rodríguez-Castañeda C.A., Pascoe-Sussoni J.E., Castelo-González O.A., Menchaca-Campos E.C., Escorcia-García J., Hu H. (2019). Optically uniform thin films of mesoporous TiO_2_ for perovskite solar cell applications. Opt. Mater..

[55] Hassan R.M., Moustafa S., Abd-Elnaiem A.M. (2020). Optimization of the linear and nonlinear optical properties of amorphous As 30 Te 69 Ga 1 thin films by the annealing process. J. Mater. Sci. Mater. Electron..

[56] Fox M. (2010). Optical Properties of Solids.

[57] El-naggar A.M., Heiba Z.K., Kamal A.M., Altowairqi Y., Mohamed M.B. (2022). Impact of loading PVA/CMC/PVP blend with CdS_0.9_M_0.1_ non-stoichiometrically doped by transition metals (M). Opt. Mater..

[58] El-naggar A.M., Heiba Z.K., Kamal A.M., Lakshminarayana G., Abd-Elkader O.H., Mohamed M.B. (2022). Preparation of PVA/CMC/PVP blended polymer loaded with ZnS_1-x_Cu_x_; investigation of structural and linear/nonlinear optical properties. Opt. Mater..

[59] Lyu M., Lee D.K., Park N.G. (2020). Effect of alkaline earth metal chloride additives BCl_2_ (B = Mg, Ca, Sr and Ba) on the photovoltaic performance of FAPbI_3_ based perovskite solar cells. Nanoscale Horiz..

[60] Mohammed M.K., Jabir M.S., Abdulzahraa H.G., Mohammed S.H., Al-Azzawi W.K., Ahmed D.S., Singh S., Kumar A., Asaithambi S., Shekargoftar M. (2022). Introduction of cadmium chloride additive to improve the performance and stability of perovskite solar cells. RSC Adv..

[61] Manjunatha K.N., Paul S. (2015). Investigation of optical properties of nickel oxide thin films deposited on different substrates. Appl. Surf. Sci..

[62] van Gorkom B.T., van der Pol T.P.A., Datta K., Wienk M.M., Janssen R.A.J. (2022). Revealing defective interfaces in perovskite solar cells from highly sensitive sub-bandgap photocurrent spectroscopy using optical cavities. Nat. Commun..

[63] Sharma P., El-Bana M.S., Fouad S.S., Sharma V. (2016). Effect of compositional dependence on physical and optical parameters of Te_17_Se_83−x_Bi_x_ glassy system. J. Alloy. Compd..

[64] Penn D.R. (1962). Wave-number-dependent dielectric function of semiconductors. Phys. Rev..

[65] Abuelwafa A.A., Alsoghier H.M., Elnobi S., Dongol M., Soga T. (2021). Quantum computational, linear and non-linear optical properties of spin-coated nickel (II)-tetraphenylporphyrin/FTO thin films. Optik.

[66] Ge M., Yang X., Cai B., Pan S., Cui H., Zhang T., Ji W. (2021). Naphthylmethylamine post-treatment of MAPbI_3_ perovskite solar cells with simultaneous defect passivation and stability improvement. Sol. Energy.

[67] Peng J., Wu Y., Ye W., Jacobs D.A., Shen H., Fu X., Wan Y., Wu N., Barugkin C., Nguyen H.T. (2017). Interface passivation using ultrathin polymer–fullerene films for high-efficiency perovskite solar cells with negligible hysteresis. Energy Environ. Sci..

[68] Peng J., Khan J.I., Liu W., Ugur E., Duong T., Wu Y., Shen H., Wang K., Dang H., Aydin E. (2018). A universal double-side passivation for high open-circuit voltage in perovskite solar cells: Role of carbonyl groups in poly (methyl methacrylate). Adv. Energy Mater..

