# Correction: Osman et al. Development of Perovskite (MACl)_0.33_FA_0.99_MA_0.01_Pb(I_0.99_Br_0.01_)_3_ Solar Cells via n-Octylammonium Iodide Surface Passivation. *Nanomaterials* 2023, *13*, 1492

**DOI:** 10.3390/nano16140857

**Published:** 2026-07-13

**Authors:** M. M. Osman, A. M. El-naggar, A. Q. Alanazi, A. M. Aldhafiri, A. A. Albassam

**Affiliations:** 1 Research Chair of Exploitation of Renewable Energy Applications in Saudi Arabia, Physics & Astronomy Department, College of Science, King Saud University, P.O. Box 2455, Riyadh 11451, Saudi Arabia; aabassam@ksu.edu.sa; 2 Physics Department, Faculty of Science (Boys Branch), Al-Azhar University, Cairo 11884, Egypt; 3 National Center for Renewable Energy Technology, KACST, P.O. Box 2455, Riyadh 11442, Saudi Arabia; aqalanazi@kacst.edu.sa; 4 Physics & Astronomy Department, College of Science, King Saud University, P.O. Box 2455, Riyadh 11451, Saudi Arabia; adhafiri@ksu.edu.sa

In the original publication [1], there were mistakes with references 56 and 57. Concerns were raised on this matter. The authors would like to replace the references with the following ones:

56. Fox, M. *Optical Properties of Solids*; Oxford University Press: Oxford, UK, 2010.

57. van Gorkom, B.T.; van der Pol, T.P.A.; Datta, K.; Wienk, M.M.; Janssen, R.A.J. Revealing defective interfaces in perovskite solar cells from highly sensitive sub-bandgap photocurrent spectroscopy using optical cavities. *Nat. Commun.* **2022**, *13*, 349. https://doi.org/10.1038/s41467-021-27560-6.

In Section 3, Results and Discussion, Paragraph 6, the following sentence has been corrected from “An electron in molecules moves from the highest occupied molecular orbital (HOMO) to the lowest unoccupied molecular orbital (LUMO) when electromagnetic radiation is absorbed [56,57].” To “An electron in molecules moves from the highest occupied molecular orbital (HOMO) to the lowest unoccupied molecular orbital (LUMO) when electromagnetic radiation is absorbed [56].”

As a result of these changes, the citation originally numbered as [57] has been removed, and the replacement reference has been renumbered as [62] to maintain the sequential order of the reference list.

With these corrections, the order of the references has been adjusted accordingly.

The authors state that the scientific conclusions are unaffected. This correction was approved by the Academic Editor. The original publication has also been updated.
